# Safety of artemether-lumefantrine in pregnant women with malaria: results of a prospective cohort study in Zambia

**DOI:** 10.1186/1475-2875-9-249

**Published:** 2010-09-01

**Authors:** Christine Manyando, Rhoda Mkandawire, Lwipa Puma, Moses Sinkala, Evans Mpabalwani, Eric Njunju, Melba Gomes, Isabela Ribeiro, Verena Walter, Mailis Virtanen, Raymond Schlienger, Marc Cousin, Miriam Chipimo, Frank M Sullivan

**Affiliations:** 1 Tropical Diseases Research Centre, Ndola, Zambia; 2 District Health Office, Choma, Zambia; 3 District Health Office, Ndola, Zambia; 4 District Health Office, Lusaka, Zambia; 5 Department of Pediatrics and Child Health, University Teaching Hospital, Lusaka, Zambia; 6 World Health Organization, Geneva, Switzerland; 7 Novartis Pharma AG, Basel, Switzerland; 8 UNICEF, Lilongwe, Malawi; 9 Former Senior Lecturer, Department of Pharmacology and Toxicology, United Medical Schools of Guy's and St Thomas' Hospitals, University of London, London, UK

## Abstract

**Background:**

Safety data regarding exposure to artemisinin-based combination therapy in pregnancy are limited. This prospective cohort study conducted in Zambia evaluated the safety of artemether-lumefantrine (AL) in pregnant women with malaria.

**Methods:**

Pregnant women attending antenatal clinics were assigned to groups based on the drug used to treat their most recent malaria episode (AL vs. sulphadoxine-pyrimethamine, SP). Safety was assessed using standard and pregnancy-specific parameters. Post-delivery follow-up was six weeks for mothers and 12 months for live births. Primary outcome was perinatal mortality (stillbirth or neonatal death within seven days after birth).

**Results:**

Data from 1,001 pregnant women (AL n = 495; SP n = 506) and 933 newborns (AL n = 466; SP n = 467) showed: perinatal mortality (AL 4.2%; SP 5.0%), comprised of early neonatal mortality (each group 2.3%), stillbirths (AL 1.9%; SP 2.7%); preterm deliveries (AL 14.1%; SP 17.4% of foetuses); and gestational age-adjusted low birth weight (AL 9.0%; SP 7.7%). Infant birth defect incidence was 1.8% AL and 1.6% SP, excluding umbilical hernia. Abortions prior to antenatal care could not be determined: abortion occurred in 4.5% of women treated with AL during their first trimester; none were reported in the 133 women exposed to SP and/or quinine during their first trimester. Overall development (including neurological assessment) was similar in both groups.

**Conclusions:**

These data suggest that exposure to AL in pregnancy, including first trimester, is not associated with particular safety risks in terms of perinatal mortality, malformations, or developmental impairment. However, more data are required on AL use during the first trimester.

## Background

More than 30 million women become pregnant each year in malaria-endemic areas of sub-Saharan Africa [1]. Malaria during pregnancy causes adverse outcomes, including abortion, anaemia, and low infant birth weight, with the latter complication due to foetal growth restriction and preterm delivery [2,3]. An important preventative strategy in highly endemic areas is intermittent preventive treatment (IPT) with sulphadoxine-pyrimethamine (SP) [4], and as SP becomes less effective with increasing *Plasmodium falciparum *resistance [5], effective treatment during pregnancy becomes more important.

The World Health Organization (WHO) recommends artemisinin-based combination therapy (ACT) as first-line treatment of *P. falciparum *malaria [6]. These guidelines state that artemisinin derivatives should be used to treat uncomplicated falciparum malaria in the second and third trimesters of pregnancy [6,7], but that they should not be used in the first trimester unless they are the only treatment available [6,7], or if the patient's life is threatened, or if treatment with quinine plus clindamycin has failed [6]. The guidelines take into account the greater confidence regarding the safety of ACT exposure in the second and third trimesters [6-11], and the scarcity of data on first trimester exposure versus the risk of embryotoxicity or death [7,12,13].

Artemether-lumefantrine (AL; Coartem^®^, Novartis Pharma AG) is currently the most widely used ACT for acute, uncomplicated *P. falciparum *malaria. Zambia was one of the first countries in Africa to replace SP with AL as first-line therapy for malaria treatment, which occurred in 2004 [14]. Here, the results of a Zambian multi-centre, prospective cohort study to evaluate the safety of AL used to treat symptomatic malaria in pregnancy are described.

## Methods

### Study population

The study was carried out in Zambia at four antenatal clinics in the districts of Choma, Ndola and Lusaka. Data were collected between October 2004 and July 2008. Pregnant women were eligible for inclusion if they had received AL or SP for the treatment of malaria. Diagnosis was clinically or parasitologically confirmed. SP was the standard anti-malarial treatment during pregnancy and also used for IPT. Pregnant women were assigned to exposure groups according to the treatment received for the "index episode", defined as the most recently treated malaria episode prior to study entry.

### Study objectives and procedures

The primary endpoint was the incidence of perinatal mortality (stillbirth or neonatal death within 7 days of birth). Secondary outcome measures were gestational age at delivery and birth weight adjusted for gestational age. In the absence of data on birth weight adjusted for gestational age from Zambia, the Zimbabwean birth weight data adjusted for gestational age as published by Munjanja & Masona in 1990 [15] was used. A newborn was considered to have low birth weight if the observed birth weight was lower than the corresponding 5^th ^percentile of the Zimbabwean birth weight, according to gestational age in keeping with international standards. In addition, the following exploratory endpoints were assessed: frequency of spontaneous abortion, preterm delivery, neonatal mortality, maternal mortality, major and minor birth defects, and infant development.

Women attending the antenatal clinic were enrolled if they reported receiving treatment with either AL (20 mg artemether and 120 mg lumefantrine) or SP (1500 mg sulphadoxine plus 75 mg pyrimethamine) according to label recommendations. Exposure was verified by documentation from their outpatient (clinic) files, which also detailed diagnostic procedures performed, and dosage of any concomitant medication given. As per government policy, women received SP, at the same doses as described above, during the second or third trimester as IPT. Treatment with any drug, including anti-malarials, prior to or after the index episode was recorded.

Women visited the antenatal clinic for assessment of safety parameters at baseline/enrolment, four weeks post-enrolment, four weeks pre-delivery, at delivery, and at six weeks post-delivery. Infants were followed up at six weeks, 14 weeks, and at 12 months after birth.

Safety assessments included monitoring and recording all adverse events (AEs) and serious adverse events (SAEs) up to six weeks after delivery. Pregnancy-specific assessments included rates of perinatal mortality defined as stillbirth (>28 weeks gestation) and early neonatal death (within seven days of birth), neonatal mortality (≤28 days of birth), maternal mortality (up to six weeks post-delivery), spontaneous abortion (≤28 weeks gestation), stillbirth, preterm delivery (≤37 completed weeks), incidence of low birth weight, gestational age at delivery (estimated from the last menstrual period [LMP], or by a developmental score [Dubowitz assessment] [16], if the LMP was unknown), and incidence of major and minor birth defects. Concomitant infections were recorded. Minor/major birth malformations were documented using a checklist. Neurodevelopmental assessment was performed at 14 weeks and 12 months after birth. The development of infants was assessed by the investigators either through a general assessment (e.g. smiling, lifting head, sitting unsupported, standing without assistance, crawling) or the Shoklo neurodevelopment assessment [17], or both.

This study was designed, implemented, and reported in accordance with ICH Good Clinical Practice, applicable local regulations, and the Declaration of Helsinki. The study protocol was approved by the local Ethics Review Committee of the Tropical Diseases Research Centre, Zambia, and WHO Ethics Review Committee, Geneva. All participants, or their parent/guardian (if the subject was a minor), gave written or finger-marked informed consent before study entry.

An independent Study Advisory Committee (SAC) reviewed unblinded data for 677 women and 392 infants on four occasions during the study and all data at the end of the study.

### Statistical analysis

Perinatal mortality was summarized by exposure (AL or SP) for the index malaria episode, together with traditional asymptotic as well as Pearson-Clopper one-sample two-sided 95% confidence intervals (CI). No formal testing for statistical significance between the two exposure groups was performed; instead, 95% CIs for the difference in proportions between the groups were constructed using traditional asymptotic methods and the Wilson score method without continuity correction. The odds ratio (with two-sided 95% CI) was calculated for perinatal mortality. Women who discontinued the study prior to delivery, or who had a spontaneous abortion were not taken into account in the primary analysis. However, the robustness of the results was assessed by supportive analyses applying multiple missing data imputation techniques [18].

The propensity score technique assessing pre-defined baseline factors (five pregnancy-related and 10 disease-related factors) was used to mitigate the potential channelling effect due to non-random assignment of therapy [19]. With the targeted sample size of 500 patients in each group, a two-sided 95% CI for precision on observed perinatal mortality (primary endpoint) would vary between 3% and 7% for an assumed background incidence of 5% in the Zambian population [20]. This precision of estimation was deemed satisfactory for a pilot study of this type. All data analyses were carried out according to a pre-established analysis plan.

## Results

### Demographics and clinical characteristics

**Figure 1:**
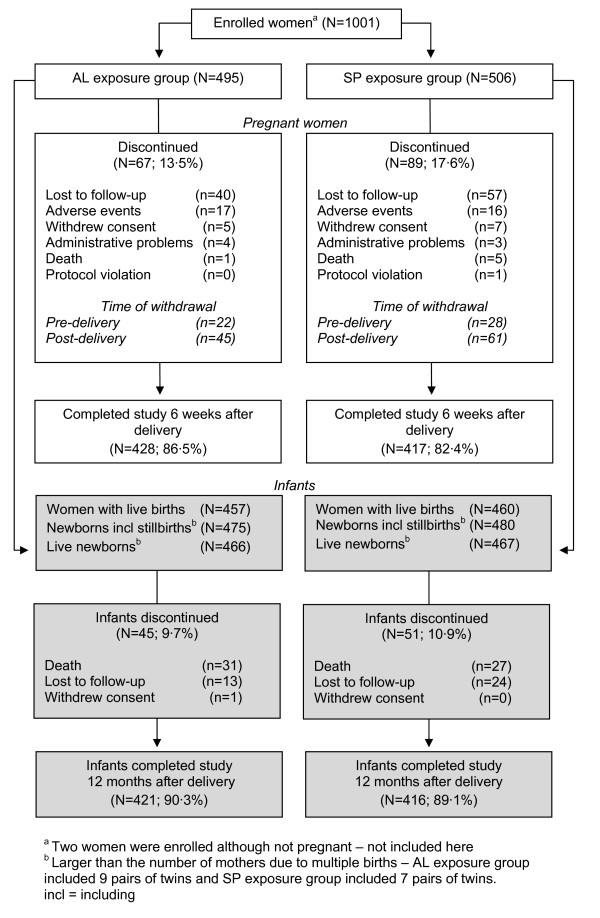
**Participant flowchart**. AL exposure group 9 pairs of twins; SP exposure group 7 pairs of twins.

A total of 1,001 pregnant women were enrolled, of whom 84.4% (845/1001) completed the study to six weeks after delivery (Figure 1). Demographic and clinical characteristics are shown in Table 1. Among the women enrolled, 98.9% had received at least some degree of schooling at the primary level and 52.2% had received some degree of secondary education.

**Table 1:** Demographic and clinical characteristics of exposure groups at enrolment and anti-malarial treatment during current pregnancy

	Exposure groups*		Total
	
	Artemether-lumefantrine (N = 495)	Sulphadoxine-pyrimethamine (N = 506)	(N = 1001)
**Age - n (%)**			

<20 years	79 (16.0)	81 (16.0)	160 (16.0)
20-24 years	167 (33.7)	151 (29.8)	318 (31.8)
25-29 years	144 (29.1)	148 (29.2)	292 (29.2)
30-39 years	101 (20.4)	119 (23.5)	220 (22.0)
≥ 40 years	4 (0.8)	7 (1.4)	11 (1.1)

**Time of index malaria episode relative to LMP - n (%)**			

Before LMP	0 (0)	2 (0.4)	2 (0.2)
Date of LMP to 12 weeks	145 (29.3)	115 (22.7)	260 (26.0)
>12 to 24 weeks	169 (34.1)	205 (40.5)	374 (37.4)
>24 weeks	162 (32.7)	161 (31.8)	323 (32.3)
LMP incomplete/unknown	19 (3.8)	23 (4.5)	42 (4.2)

**Treatment courses during first trimester including IPT with SP - n (%)**			

Only AL	142 (28.7)	5 (1.0)	147 (14.7)
Only SP	7 (1.4)	120 (23.7)	127 (12.7)
AL and SP	8 (1.6)	1 (0.2)	9 (0.9)
SP plus quinine	0 (0)	2 (0.4)	2 (0.2)
Only quinine	2 (0.4)	2 (0.4)	4 (0.4)
None (including unknown LMP date)	336 (67.9)	376 (74.3)	712 (71.1)

Treatment received during current pregnancy **- n (%)	**(N = 473)**	**(N = 478)**	**(N = 951)**

Only AL or SP	51 (10.8)	449 (93.9)	500 (52.6)
Both AL and SP	412 (87.1)	13 (2.7)	425 (44.7)
AL or SP plus quinine	1 (0.2)	13 (2.7)	14 (1.5)
Both AL and SP plus quinine	9 (1.9)	3 (0.6)	12 (1.3)

**Alcohol status (current)**	**(N = 495)**	**(N = 506)**	**(N = 1001)**

Non-drinker	441 (89.1)	437 (86.4)	878 (87.7)
Drinker	14 (2.8)	17 (3.4)	31 (3.1)
Unknown	40 (8.1)	52 (10.3)	92 (9.2)

**Alcohol intake frequency**			

Low	11 (2.2)	11 (2.2)	22 (2.2)
Medium	3 (0.6)	5 (1.0)	8 (0.8)
High	0	1 (0.2)	1 (0.1)

**Tobacco smoking history (current)**			

Non-smoker	455 (91.9)	454 (89.7)	909 (90.8)

Smoker	0	0	0
Unknown	40 (8.1)	52 (10.3)	92 (9.2)

Enrolled population: pregnant women who gave informed consent.

*Exposure groups represent the treatment given to the pregnant woman for the falciparum malaria episode that had occurred most recently prior to enrolment (index episode).

** Data from women with known delivery status.

AL = artemether-lumefantrine

IPT = intermittent preventive treatment (with SP)

LMP = last menstrual period

SP = sulphadoxine-pyrimethamine

The index episode was predominantly diagnosed based on symptoms: malaria was unconfirmed in 82.0% of the AL and 87.2% of the SP exposure groups. Details of anti-malarial treatment received during the current pregnancy are shown in Table 1. The majority of women with known delivery status in the AL group (412/473; 87.1%) had received both AL and SP (as IPT) during the current pregnancy. Conversely, most women in the SP group had received only SP for treatment (449/478; 93.9%) and IPT prophylaxis in the 2^nd ^and 3^rd ^trimesters, in accordance with National Health policy in Zambia; a single dose of 1500 mg of sulphadoxine plus 75 mg pyrimethamine was used for treatment or prophylaxis. In total, 2.7% (13/478) of women in the SP group were exposed to both AL and SP. Most women received two to four courses of anti-malarial treatment during the pregnancy, including IPT. A small proportion of women in each group (AL 2.1%; SP 3.3%) were also exposed to quinine. Approximately 70% of enrolled women had taken their first anti-malarial treatment 12 weeks after their LMP. In total, 156 women (150/495 [30.3%] from the AL group, plus 6 from the SP group) received AL treatment during the first trimester, while 138 women (123/506 [24.3%] from the SP group, plus 15 from the AL group) received SP treatment during the first trimester.

All patients reported taking the full prescribed course of anti-malarial treatment, and very few (<0.4%) reported vomiting the medication. More than 95% of all enrolled women received at least one concomitant medication (other than anti-malarial therapy), most commonly folic acid and oral iron preparations. All women were counselled for HIV testing. Approximately 30% of women in the AL group and 38% in the SP exposure group were tested for HIV: overall, 22% of women with a test result were reported as HIV-positive.

### Pregnancy outcomes

A summary of pregnancy outcomes is shown in Table 2. Overall perinatal mortality was similar for both exposure groups (Table 3) (AL 4.2% [asymptotic 95% CI 2.4 - 6.0]; SP 5.0% [asymptotic 95% CI 3.1 - 6.9]; odds ratio [AL versus SP] 0.84 [asymptotic 95% CI 0.45 - 1.53]). First trimester perinatal mortality is reported in detail below. Subgroup analyses on perinatal mortality did not show any significant between-group differences.

**Table 2:** Pregnancy outcome by exposure group

Pregnancy outcome - n (%)	Artemether-lumefantrine (N = 504)	Sulphadoxine-pyrimethamine (N = 516)
Abortion (≤28 weeks of gestation)	7* (1.4)	8** (1.6)
Stillbirth (>28 weeks of gestation)	9 (1.8)	13 (2.5)
Preterm delivery (<37 completed weeks of gestation)	71 (14.1)	90 (17.4)
Full term delivery (≥37 completed weeks of gestation)	395 (78.4)	377 (73.1)
Unknown (mother withdrawn prior to delivery)	22 (4.4)	28 (5.4)

Enrolled population: newborns, stillbirths, and aborted foetuses or pregnant women who were withdrawn prior to delivery.

* All first trimester exposures.

**5 abortions: one woman with triplets, one with twins - none had first trimester exposure.

One patient from the AL group had an ectopic pregnancy with laparotomy performed to remove the foetus. This patient has pregnancy outcome "unknown" as she was withdrawn due to an adverse event (ectopic pregnancy) prior to "delivery".

**Table 3:** Perinatal mortality (stillbirths plus neonatal deaths) by exposure group

	Artemether-lumefantrine (N = 475)			Sulphadoxine-pyrimethamine (N = 480)	
**Perinatal mortality (primary outcome) - n (%)**	20 (4.2)			24 (5.0)	

Asymptotic 95% CI	2.4 - 6.0			3.1 - 6.9	
Pearson-Clopper 95% CI	2.6 - 6.4			3.2 - 7.3	
Stillbirth - n (%)	9 (1.9)			13 (2.7)	
Neonatal death ≤7 days after birth - n (%)	11 (2.3)			11 (2.3)	

	Exposure to anti-malarial agent(s) during first trimester*				

	**AL only (N = 135)**	**AL + SP (N = 7)**	**SP or/and quinine (N = 129)**	**None (N = 644)**	**Unknown (N = 40)**

**Perinatal mortality - n (%)**	**6 (4.4)**	**1 (14.3)**	**5 (3.9)**	**31 (4.8)**	**1 (2.5)**

Stillbirth (>28 weeks gestation)	2 (1.5)	0 (0)	3 (2.3)	17 (2.6)	0 (0)
Death ≤7 days after birth	4 (3.0)	1 (14.3)	2 (1.6)	14 (2.2)	1 (2.5)

Enrolled population: newborns and stillbirths.

Perinatal mortality: stillbirth or early neonatal death within 7 days after birth.

AL = artemether-lumefantrine; CI = confidence interval; SP = sulphadoxine-pyrimethamine.

* Data from newborns and stillbirths with known delivery status.

Perinatal mortality status was missing for 6.2% of women due to abortion (1.2%) or withdrawal prior to delivery (5.0%). However, multiple imputation analyses demonstrated that the results were robust with respect to missing data.

Women treated with AL for the index episode had a higher frequency of confirmed malaria in comparison with women treated with SP. An analysis to correct for any possible channelling bias showed no effect on perinatal mortality due to more AL patients having confirmed malaria (adjusted odds ratio of 0.79 [95% Wald CI 0.43 - 1.46] versus the observed unadjusted odds ratio of 0.84 [asymptotic 95% CI 0.45 - 1.53]).

Stillbirths occurred in 1.9% of the AL group and in 2.7% of the SP group. Of the nine mothers who had stillbirths in the AL group, AL was the last exposure in three cases; eight women had been exposed to SP for IPT, six were exposed after the AL-treated index episode, and two before it. Thirteen women in the SP group had stillbirths: in five cases, SP was the last exposure (as treatment for the index episode); six of the thirteen women had IPT; and two women were treated with quinine for additional episodes of malaria.

Early neonatal death was similar for each exposure group (2.3% for each group). In the AL group, four of the 11 neonatal deaths occurred after delivery at home. All 11 neonatal deaths in the SP group occurred after delivery at a hospital or health centre.

There were seven cases of abortion in the AL group, all occurring in women who were exposed to AL during their first trimester (further details below); the last anti-malarial exposure was AL (index episode) in six cases, and in the remaining case SP was given for treatment of a malaria episode occurring after the index episode. Eight abortions occurred in five women in the SP group, all second or third trimester exposures (one was carrying triplets, another was carrying twins): the last anti-malarial exposure was SP (index episode) in one case, SP (IPT) in three cases, and quinine (additional malaria episode) in the remaining case.

### Infant outcomes at birth

Infant outcomes are summarized in Table 4. Low birth weight according to gestational age occurred in 9.0% of the AL and 7.7% of the SP group. Mean and median birth weights, head circumference, body length, and sex distribution were similar in both exposure groups. Mean and median gestational ages determined using LMP were comparable for the two groups (39.0 weeks each), as were the values calculated from the Dubowitz test (mean/median: AL group 37.6/37.7 weeks; SP group 38.2/38.1 weeks).

**Table 4:** Gestational age and weight of infants at birth by exposure group

	Artemether-lumefantrine (N = 466)	Sulphadoxine-pyrimethamine (N = 467)
**Gestational age at delivery, determined by LMP**		

Number of observations	448	445
Mean/Median weeks	39.0/39.0	38.9/39.0
Distribution - n (%)		
≤28 weeks	5 (1.1)	2 (0.4)
>28 to <37 weeks	58 (12.4)	85 (18.2)
≥37 weeks	385 (82.6)	358 (76.7)
Missing	18 (3.9)	22 (4.7)

**Birth weight - g**		

Number of observations	448	444
Mean (SD)	3058.3 (515.96)	3039.2 (495.26)
Median (Range)	3000 (900 - 4700)	3000 (1300 - 4500)

Enrolled population: newborn infants.

LMP = last menstrual period

Patients with a missing birth weight or a missing gestational age were not taken into account in this Table.

Birth defects in infants were detected by midwives in 6.5% of the AL group and 4.1% of the SP group (Table 5). The between-group difference was largely due to a higher rate of umbilical hernia in the AL group (AL 4.7%; SP 2.7%). The majority of these had resolved by the 12 month evaluation (Table 6). Excluding umbilical hernia, which is a common reversible defect in African children [21,22], the infant birth defect frequency was 1.8% for AL and 1.6% for SP. There was no pattern of distribution of any birth defect in either exposure group. In the AL group, multiple birth defects were reported in two infants: one had trisomy 21, the other had trisomy 18. Polydactyly was reported in seven infants and occurred in a similar proportion in each group (AL 0.7%; SP 0.9%).

**Table 5:** Birth defects reported from the time of delivery to 14 weeks by exposure group

	Artemether-lumefantrine (N = 466)	Sulphadoxine-pyrimethamine (N = 467)
**Total number infants with malformations**^a ^**- n (%)**	29/449^b ^(6.5)	18/444^b ^(4.1)
**Diagnosis of congenital anomaly/birth defect**		
Umbilical hernia	21 (4.7)	12 (2.7)
Polydactyly	3 (0.7)	4 (0.9)
Dermal cyst	0 (-)	1 (0.2)
Ear malformation	0 (-)	1 (0.2)
Genitourinary system: small labia^c^	1 (0.2)	0 (-)
Hair: lanugo^c^	1 (0.2)	0 (-)
Inguinal hernia	0 (-)	1 (0.2)
Limbs: hyperextensibility of joint	1 (0.2)	0 (-)
Nose: small^c^	1 (0.2)	0 (-)
Skin hyperpigmentation	1 (0.2)	0 (-)
Trisomy 18^d^	1 (0.2)	0 (-)
Trisomy 21^e^	1 (0.2)	0 (-)

Enrolled population: newborn infants.

No distinction was made between major and minor birth defects on the case report form.

^a^Any malformation = any finding other than normal or any diagnosis of congenital anomaly/birth defect reported.

^b^Number of newborn infants with assessment performed at least once at delivery, 6 weeks, or 14 weeks post-delivery. No assessments were available for 17 infants in the AL group and 23 in the SP group.

^c^Reported in the same patient.

^d^Reported malformations: Ears - low set; Mouth - high arched palate; Cranium - microencephaly/anencephaly; Limbs - clenched with overlapping fingers.

^e^Reported malformations: Ears - low set; Mouth - high arched palate; CVS murmur; Chest - short sternum; Cranium - low occiput; Eyes - upward slanting; Mouth - protruding tongue; Neck - short; Nose - low nasal bridge.

In the SP group, one child was reported to have had umbilical hernia and polydactyly.

**Table 6:** Birth defects reported from time of delivery until 14 weeks after birth, according to anti-malarial drug exposure during the first trimester

	Exposure to anti-malarial(s) during first trimester				
	
	AL only (N = 133)	AL plus SP (N = 7)	SP or/and quinine (N = 126)	None (N = 627)	Unknown (N = 40)
Total number of infants with malformations (%)	9/130 (6.9)	0/7 (-)	8/121 (6.6)	27/596 (4.5)	3/39 (7.7)
Umbilical hernia^4^	8 (6.2)	0 (-)	5 (4.1)	20 (3.4)	0 (-)
Polydactyly	0 (-)	0 (-)	1 (0.8)	4 (0.7)	2 (5.1)
Dermal cyst	0(-)	0 (-)	1 (0.8)	0 (-)	0 (-)
Ear malformation	0 (-)	0 (-)	1 (0.8)	0 (-)	0 (-)
Genitourinary system: small labia^1^	1 (0.8)	0 (-)	0 (-)	0 (-)	0 (-)
Hair: lanugo^1^	1 (0.8)	0 (-)	0 (-)	0 (-)	0 (-)
Inguinal hernia	0 (-)	0 (-)	0 (-)	1 (0.2)	0 (-)
Limbs: hyperextensibility of joint	0 (-)	0 (-)	0 (-)	0 (-)	1 (2.6)
Nose: small^1^	1 (0.8)	0 (-)	0 (-)	0 (-)	0 (-)
Skin hyperpigmentation	0 (-)	0 (-)	0 (-)	1 (0.2)	0 (-)
Trisomy 18^2^	0 (-)	0 (-)	0 (-)	1 (0.2)	0 (-)
Trisomy 21^3^	0 (-)	0 (-)	0 (-)	1 (0.2)	0 (-)

Enrolled population: newborns.

^1 ^All of these malformations were reported in the same patient.

^2 ^Reported malformations in this patient: Ears - low set; Mouth - high arched palate; Cranium microencephaly/anencephaly; Limbs - clenched with overlapping fingers.

^3 ^Reported malformations in this patient: Ears - low set; Mouth - high arched palate; CVS murmur; Chest - short sternum; Cranium: flat occiput; Eyes - upward slanting; Mouth: protruding tongue; Neck - short; Nose - low nasal bridge.

^4 ^No umbilical hernia was detected at birth. Of 21 cases reported in the AL exposure group, 12 detected at week 6 were resolved by week 14. All remaining umbilical hernias had resolved by 12 months except for one infant who died prior to 12 months from a febrile illness. The mother of one infant with an umbilical hernia had received quinine during the first trimester of pregnancy, before AL treatment of the index episode (not in the first trimester) and another had received SP during the first trimester. In eight additional cases, the mother had received AL treatment in the first trimester, in each case to treat the index episode of malaria. In the SP group, among 12 infants with umbilical hernias, 7 had resolved by week 14, the remainder by month 12, with the exception of 1 case lost to follow-up, and one infant who died from pneumonia.

### Infant outcomes and developmental assessments from birth to 12 months

Mean and median height and weight were very similar in both groups at birth, 14 weeks, and at 12 months after delivery (data not shown). The general developmental assessment in 833 infants (427 AL; 406 SP) at 14 weeks (smiling, lifting head) and at 12 months (sitting unsupported, standing without assistance, crawling) was also similar for each group, and virtually all infants met their expected developmental milestones. In addition, infants of mothers only exposed to AL or SP compared well with the overall group.

Data from the Shoklo neurodevelopmental evaluation, performed in 530 infants (298 AL; 232 SP), revealed very similar mean and median values for each exposure group. Tone and behaviour scores were similar at 14 weeks and 12 months, but motor milestone and coordination scores increased considerably over the study period. No differences in Shoklo neurodevelopment scores were observed in infants of mothers who had been exposed only to AL or to SP: more than 98% of infants assessed achieved a score of "good" or "excellent" at 14 weeks and at 12 months.

Neonatal mortality rate (death within 28 days of birth) was 3.0% in each group. In many cases the cause of death was unknown and the most common reported causes were prematurity and asphyxia.

### Exposure to anti-malarials in the first trimester (exploratory analyses)

A total of 156 women (150/495 [30.3%] from the AL plus 6 from the SP group) received AL treatment during the first trimester, primarily for treatment of the index episode (Table 1). Foetuses of women exposed to AL only during the first trimester had a similar rate of full-term delivery to those who had no first trimester anti-malarial exposure (AL alone: 75.3% [113/150]; none: 76.5% [523/684]). The rate of preterm delivery was highest in foetuses of women exposed to SP and/or quinine during the first trimester (SP and/or quinine: 20.7% [28/135]; AL alone 13.3% [20/150]; none 15.2% [104/684]) (Table 7).

**Table 7:** Pregnancy outcome by exposure group, according to anti-malarial drug exposure during the first trimester

	Exposure to anti-malarial(s) during first trimester				
	**AL only** **(N = 150)**	**AL plus SP** **(N = 9)**	**SP or/and quinine** **(N = 135)**	**None** **(N = 684)**	**Unknown** **(N = 42)**

**Pregnancy outcome - n (%)**					

Abortion (≤28 weeks)	4 (2.7)	2 (22.2)	0 (-)	8* (1.2)	1** (2.4)
Stillbirth (>28 weeks)	2 (1.3)	0 (-)	3 (2.2)	17 (2.5)	0 (-)
Preterm delivery (<37 weeks)	20 (13.3)	1 (11.1)	28 (20.7)	104 (15.2)	8 (19.0)
Full term delivery (≥37 weeks)	113 (75.3)	6 (66.7)	98 (72.6)	523 (76.5)	32 (76.2)
Unknown (mother withdrawn prior to delivery)	11 (7.3)	0 (-)	6 (4.4)	32 (4.7)	1 (2.4)

Enrolled population: newborns, stillbirths and aborted foetuses

AL = artemether-lumefantrine; SP = sulphadoxine-pyrimethamine

*5 abortions: one woman with triplets, one with twins

** Incomplete date of last menstrual period, therefore, counted as unknown first trimester exposure (a woman who was likely to have taken AL during the first trimester)

A total of 159 foetuses were exposed to AL during the first trimester, either AL only (n = 150) or AL plus SP (n = 9) (Table 7). Six abortions occurred in this population (AL alone: 4 cases; AL plus SP: 2 cases) giving a rate of 3.8% (Table 8). One further abortion occurred in a woman who was likely to have taken AL during the first trimester (incomplete LMP date). Confounding factors were present in four cases; one had syphilis, two had multiple malarial episodes, and one received concomitant treatment with salbutamol and AL for a threatened abortion. In 135 foetuses exposed to SP and/or quinine during the first trimester, no abortions were reported.

**Table 8:** Abortion occurring in women treated with artemether-lumefantrine

Case	Time of abortion (gestation week)	Time of AL treatment (gestation week)	Reason for AL treatment	Comments
A	13	9	Index episode	Syphilis at Week 10
B	10	6	Index episode	-
C	18	6	Index episode	-
D	15	7	Index episode	Viable embryo at Week 11 on ultrasound
E	27^a^	6 and 16	Index episode	3 malaria episodes at Weeks 6, 11 (received SP), and 16
F	22	10	Non-index episode	2 previous spontaneous abortions; 3 malaria episodes at Weeks 7 (received SP), 10, and 22 (received SP); at Week 20 severe respiratory tract infection, anaemia, oral candidiasis, and immunosuppression (suspected HIV positive): spontaneous abortion at Week 22, followed by maternal death 24 hours later
G^b^	6 days after AL treatment	Between Weeks 6 and 9	Index episode	Treatment with salbutamol for threatened abortion during AL treatment

All cases of abortion occurred in women who received AL during the first trimester; cases A-D, and G received AL only during the pregnancy.

Index episode: falciparum malaria episode that had occurred most recently prior to study enrolment.

AL = artemether-lumefantrine; SP = sulphadoxine-pyrimethamine

^a ^Therapeutic abortion following intrauterine death.

^b ^Incomplete LMP date, therefore, counted as unknown first trimester exposure.

Perinatal mortality was very similar whether first trimester exposure was to AL alone, or to SP and/or quinine, or if no anti-malarial therapy had been received (Table 3). Among women who received AL during the first trimester, 4.3% (6/140) newborns died within 28 days after birth compared with 3.0% (19/627) among women who received no anti-malarials and 1.6% (2/126) among women who only received SP and/or quinine during the first trimester.

Birth weight (adjusted for gestational age or not) showed no notable differences between the groups.

The incidence of malformations in babies born to mothers who received AL only in the first trimester was 6.9% (9/130); eight of these nine cases were umbilical hernias, and the remaining baby was reported as having small genital labia, lanugo, and a small nose (Table 6). The malformation rate in infants from women exposed to other anti-malarial agents was 6.6% (8/121), and 4.5% (27/596) in those with no anti-malarial exposure in the first trimester.

The Shoklo neurodevelopmental assessment of infants born to women exposed to AL in the first trimester were very similar to results for SP: 98.8% (83/84) of infants from the AL group achieved a score of "good" or "excellent" at 14 weeks, and 100% (78/78) achieved the same scores at 12 months.

### General safety outcomes

A summary of common AEs is presented in Table 9. The two groups were generally similar in their observed AE profiles, the most notable differences being the rates of premature delivery and of malaria as an AE, both of which were higher in the SP group. SAEs, most commonly complications of pregnancy and infections, occurred in 6.5% of women in the AL group and in 7.3% of those exposed to SP.

**Table 9:** Common* adverse events by exposure group

**MedDRA Primary System Organ Class**: **MedDRA Preferred Term**	Artemether-lumefantrine N = 495 n (%)	Sulphadoxine-pyrimethamine N = 506 n (%)
**Total number of patients with any adverse events**	**171 (34.5)**	**186 (36.8)**

**Blood and lymphatic system disorders**:	**7 (1.4)**	**16 (3.4)**

Anaemia	7 (1.4)	15 (3.2)

**Infections and infestations**:	**61 (12.3)**	**66 (13.0)**

Malaria	17 (3.4)	34 (6.7)
Syphilis	24 (4.8)	20 (4.0)
Respiratory tract infection	9 (1.8)	5 (1.0)
Urinary tract infection	5 (1.0)	5 (1.0)

**Nervous system disorders**:	**1 (0.2)**	**8 (1.6)**

Headache	1 (0.2)	6 (1.2)

**Pregnancy, puerperium, and perinatal conditions**:	**108 (21.8)**	**120 (23.7)**

Premature baby	69 (13.9)	87 (17.2)
Stillbirth	9 (1.8)	13 (2.6)
Cephalo-pelvic disproportion	11 (2.2)	6 (1.2)
Abortion spontaneous	6 (1.2)	5 (1.0)

Enrolled population: pregnant women who gave informed consent.

MedDRA = medical dictionary for regulatory activities

A pregnant woman with multiple occurrences of an adverse event is counted only once in the corresponding adverse event category.

*Preferred terms occurring in at least 1% of women in either exposure group are presented along with the corresponding primary system organ class.

There were six maternal deaths during the study; one death (0.2%) in the AL group (anaemia secondary to spontaneous abortion), and five deaths (1.0%) in the SP group (one from Kaposi's sarcoma, three from infections [pneumonia, sepsis, and viral encephalitis], and one from "undiagnosed illness"). Mean haemoglobin levels 4 weeks after enrolment and at delivery were 12.0 and 11.6 g/dL in the AL group, and 11.9 and 11.7 g/dL in the SP group, respectively.

The incidence of non-fatal SAEs was similar (AL 21.8% [108/495] vs. SP 23.3% [118/506]). The most frequent non-fatal SAEs were related to pregnancy and birth, specifically premature delivery (AL 13.7%; SP 17.2%), stillbirth, cephalo-pelvic disproportion, and abortion. Other SAEs included infections (AL 0.6%; SP 0.8%), of which there were three cases of severe malaria in the SP group.

## Discussion

WHO treatment guidelines exclude artemisinin compounds for use in the first trimester of pregnancy unless there is no alternative, or the mother's life is at stake [6,7]. Preclinical data have raised concerns for ACT exposure in early pregnancy [7,12,13], and yet, despite an unprecedented roll out of ACT, there are only 125 reported cases of human first trimester exposure to artemisinins, albeit with no adverse outcomes [7], a number insufficient to be reassuring regarding safety [7,23,24]. This report is based on maternal and birth outcomes in the largest series of prospectively followed ACT-exposed pregnancies, in which there were 495 women in the AL exposure group, and 156 were exposed during the first trimester. Outcomes were compared against SP, the most widely used anti-malarial agent administered during pregnancy. Furthermore, a prolonged follow up of infants permitted a thorough investigation of their development following anti-malarial exposure *in utero*.

The observed rate of perinatal mortality was 4.2% in the AL group and 5.0% in the SP group, which is comparable with other data from malaria endemic areas [25-27]. There were no differences between groups with regard to neonatal mortality, maternal mortality, rates of stillbirth, low birth weight, and infant neurological development. Preterm delivery using reported LMP dates (which are notoriously unreliable) was slightly more common in the SP group (17.4%; AL group 14.1%), but the majority of infants had birth weights consistent with full-term delivery. Anti-malarial treatment was reportedly well-tolerated. Overall, 22% of 326 women with a reported test result (i.e. approximately 7% of the women in each exposure group) had an HIV-positive test. The overall profiles for AEs and SAEs were unremarkable: besides pregnancy complications, most of the commonly reported AEs and SAEs were due to infections, which were also responsible for the majority of maternal deaths.

Women with first trimester AL exposures did not have a greater perinatal mortality rate compared with SP exposure. Infant neurodevelopmental assessments were also similar, irrespective of drug exposure in first trimester. However, there were seven abortions in 159 foetuses exposed to AL in their first trimester, and no abortions in 135 foetuses exposed to SP and/or quinine in their first trimester. In four AL-exposed abortions, the women had risk factors for abortion: two women had three malaria episodes within the first trimester, one woman had syphilis diagnosed 1 week post-AL exposure, and one woman was given salbutamol for threatened abortion concomitantly with AL therapy for malaria. Furthermore, there was no clear relationship between the time at which AL exposure occurred and the occurrence of abortion; except in one case, the abortion occurred long after anti-malarial drug ingestion. Women were mainly enrolled after their first trimester and, therefore, this study could not determine the potential number of abortions that occurred during the first trimester. This may explain the lower abortion rate observed, compared with the 12-16% spontaneous abortion rate documented [28,29]. It is difficult to draw any definite conclusions from the data, but further monitoring of first trimester exposures to AL is warranted.

The frequency of birth defects was low and the SAC did not consider that any reported defects (excluding the cases of trisomy) could be described as major. The most frequent finding was umbilical hernia (AL 4.7%; SP 2.7%) and all cases had resolved within 12 months. Umbilical hernia has been reported in rats exposed to artemisinins during gestation [30,31]. It is commonly observed in African infants (17%-23%) [21,22], is not generally considered as a "malformation" and the frequency reported here was less than previously documented [21,22]. Polydactyly, another reported malformation, is also commonly found in African infants [32,33]. Although reasons for the low incidence of major malformations in this study remain unclear, it is unlikely that major malformations (as described by van Regemorter, [34]) would have been missed. Cardiac defects were not detected as cardiac auscultation and/or echocardiograms were not routinely performed, and autopsies were not carried out on deceased infants.

With new data on more than 300 women exposed to AL (referring to index episodes) during the second and third trimester, this study substantially adds to data on the safety of artemisinins in the later stages of pregnancy in African women [35,36], confirming previous findings that AL exposure during this period is well tolerated and has no adverse outcomes for the mother or her exposed foetus.

The study had both strengths and limitations. Firstly, it was undertaken in the context of care provided at antenatal clinics in Zambia, and represents the demographic, infectious disease, and drug exposure profile of pregnant women coming for routine care. The acquisition of drug safety data in pregnancy via clinical trials is associated with various difficulties, risks and costs [37]. Regulatory authorities now recommend observational prospective designs for detecting drug exposure risks or generating margins of reassurance regarding lack of drug exposure risk during pregnancy [38,39]. This study followed these recommendations. The majority of women (>87% AL group) had received IPT with SP prior to enrolment or thereafter (only 51 women were exposed to AL alone), thereby protecting the women from malaria, but also confounding the evaluation of AL exposure. The index episode was unconfirmed by microscopy or rapid diagnostic test in most (85%) participants, which is normal clinical practice at the level of health centres in Zambia where the study was carried out, and it is likely that some of the women exposed to AL and SP may have had other febrile illnesses. This does not change the validity of the information collected, but prevents an analysis of whether the presence of malaria parasites (to which the artemisinins preferentially bind) changed any risk.

Gestational age was assessed by midwives either via LMP (the primary method indicated in the protocol) or through use of the Dubowitz test. The Dubowitz test has been shown to be a reliable method for assessment of gestational age in African neonates [40]. However, although all of the investigators received the same type and frequency of training from the same trainers to allow uniform standardized assessments, these results need to be interpreted with some caution as the neonatal assessments were conducted over a long duration of time at multiple sites.

A low incidence of major birth defects could either reflect a lower risk or weaknesses in identifying these risks [41]. On balance, however, as both groups were prospectively monitored without knowing the outcome, the safety data obtained reflect the unbiased outcomes of pregnancies exposed to multiple infections and multiple drugs, including anti-malarials, rather than the consequences of a single well-defined treatment for a single infectious episode.

## Conclusions

With increasing resistance to SP, establishing the margin of safety of ACT in pregnancy is critical. Results from this cohort study confirm that exposure to AL during later pregnancy is not associated with increased safety risks in terms of perinatal mortality, malformations, or infant neurodevelopment, but they also suggest that more safety data are required on first trimester exposure to AL and consequently for ACT in general.

## List of abbreviations used

ACT: artemisinin-based combination therapy; AE: adverse event; AL: artemether-lumefantrine; CI: confidence intervals; IPT: intermittent preventive treatment; LMP: last menstrual period; SAC: Study Advisory Committee; SAE: serious adverse event; SP: sulphadoxine-pyrimethamine; WHO: World Health Organization.

## Competing interests

CM, RM, LP, MS, EM, EN, MCh, and FMS received payments to attend meetings related to the study. VW, RS, and MCo are all full time employees of Novartis Pharma and VW and MCo hold stock ownership therein. MG is a full time employee of the WHO. For the duration of the study and manuscript preparation period, MV was a full time employee of Novartis Pharma and holds stock ownership therein. For the duration of the study, IR worked as a full-time consultant for the WHO.

## Authors' contributions

The authors of this manuscript were involved in study design, data interpretation, and the writing of the report, or in a combination of these activities. All authors had full access to study data and held equal final responsibility for the decision to submit this report for publication. All authors contributed either to the design of the study or assisted with data interpretation. CM and MV coordinated the study and supervised enrolment and patient follow-up. VW, RS, MV and CM participated in data entry, collection, and analysis of data. All authors participated in the preparation of the manuscript and approved the final version.
